# Retraction: Magnitude and associated factors of mortality among patients admitted with COVID-19 in Addis Ababa, Ethiopia

**DOI:** 10.1371/journal.pgph.0003279

**Published:** 2024-05-16

**Authors:** The PLOS Global Public Health Editors

After this article [1] was published, it came to the attention of the *PLOS Global Public Health* Editors that similar work had previously been published by the same author group in another journal [2] under the Creative Commons Attribution License [3]. Despite some differences in the text, there is substantial overlap between the two articles in the study methodology, results and conclusions.

In light of the above concerns about redundancy of this work, the *PLOS Global Public Health* Editors retract this article.

All authors either did not respond directly or could not be reached.
